# Gastroenteritis due to typhoidal *Salmonella*: a decade of observation at an urban and a rural diarrheal disease hospital in Bangladesh

**DOI:** 10.1186/1471-2334-14-435

**Published:** 2014-08-07

**Authors:** Sumon Kumar Das, Mohammod Jobayer Chisti, Mokibul Hassan Afrad, Mohammad Abdul Malek, Shahnawaz Ahmed, Farzana Ferdous, Fahmida Dil Farzana, Jui Das, KM Shahunja, Farzana Afroze, Mohammed Abdus Salam, Tahmeed Ahmed, Abu Syed Golam Faruque, Peter John Baker, Abdullah Al Mamun

**Affiliations:** Centre for Nutrition and Food Security (CNFS), International Centre for Diarrhoeal Disease Research, Bangladesh (icddr,b), Dhaka, Bangladesh; School of Population Health, The University of Queensland, Brisbane, Australia; Department of Clinical Trial and Clinical Epidemiology, Graduate School of Comprehensive Human Sciences, University of Tsukuba, Tsukuba, Ibaraki, Japan

**Keywords:** Bangladesh, Diarrhea, Rural, Typhoidal *Salmonella*, Urban

## Abstract

**Background:**

The study aimed to compare the socio-demographic, host and clinical characteristics, seasonality and antimicrobial susceptibility of Typhoidal *Salmonella (Salmonella enterica* serovar Typhi and Paratyphi) (TS) with diarrhea between urban and rural Bangladesh.

**Methods:**

Relevant information of 77/25,767 (0.30%) and 290/17,622 (1.65%) patients positive with TS (in stool) were extracted from the data archive of Diarrheal Disease Surveillance System of icddr,b (urban Dhaka and rural Matlab Hospitals respectively) during 2000–2012. Comparison group (diarrhea patients negative for TS) was randomly selected from the database (1:3 ratio). Two poisson regression models were investigated for modelling seasonal effects on the number of cases.

**Results:**

*Salmonella* Typhi was more frequently isolated in Dhaka than Matlab (57% vs. 5%, p < 0.001); while *Salmonella* Paratyphi was more frequent in Matlab than Dhaka (96% vs. 43%; p < 0.001). Fever [adj. OR-5.86 (95% CI: 2.16, 15.94)], antimicrobial use at home [5.08 (2.60, 9.90)], and fecal red blood cells [2.53 (1.38, 4.64)] were significantly associated with detection of TS in stool of patient from Dhaka. For Matlab, the correlates were, vomiting [1.88 (1.35, 2.64)], fecal macrophage [1.89 (1.29, 2.74)] in addition to fever and duration of diarrhea and antimicrobial use. At Dhaka, all *Salmonella* Typhi isolates were susceptible to ceftriaxone; while in Dhaka and Matlab however, for ciprofloxacin it was 45% and 91%, respectively. Susceptibility to chloramphenicol, ampicillin, trimethoprim-sulphamethoxazole and nalidixic acid ranged from 12%-58%. *Salmonella* Paratyphi were susceptible to ceftriaxone (99%). A significant seasonal trend and year difference (before and after 2007) for Matlab was observed (p < 0.001 for all effects). Dhaka does not show significant year or seasonal effects (p = 0.07 for years and p = 0.81 and p = 0.18 for the cos and sin components, respectively). While not significant, two seasonal peaks were observed in Dhaka (January-February and September-November); while a single peak (August-November) was observed in Matlab.

**Conclusions:**

Proportion of serovar distribution of TS and their clinical characteristics, antimicrobial susceptibility and seasonal pattern were different among diarrhea patients in urban Dhaka and rural Matlab of Bangladesh.

**Electronic supplementary material:**

The online version of this article (doi:10.1186/1471-2334-14-435) contains supplementary material, which is available to authorized users.

## Background

Typhoid and paratyphoid fever may be associated with constipation, normal bowel habit or diarrhea. There is lack of reporting on gastroenteritis due to *Salmonella enterica* serovar Typhi (*S.* Typhi) and Paratyphi (*S.* Paratyphi) globally due to their lower incidence relative to illness caused by more frequent enteric pathogens such as rotavirus, *Shigella*, non-typhoidal *Salmonella*, *Campylobacter jejuni*, *Escherichia coli* and *Vibrio cholerae* in developing countries [1–5]. Both of these *Salmonella* serovars or typhoidal *Salmonella* (TS) cause enteric fever, which may be fatal if not adequately treated. Clinical description of non-typhoidal *Salmonella* (NTS) gastroenteritis, more common in developed countries, is available [6, 7]; however, there is lack of published literatures on the clinical, host and socio-demographic characteristics of gastroenteritis due to typhoidal *Salmonella*. Moreover, the differences in the disease prevalence and outcome between urban and rural areas are not well investigated.

The International Centre for Diarrhoeal Disease Research, Bangladesh (icddr,b) maintains Diarrheal Disease Surveillance System (DDSS) at its urban Dhaka Hospital and rural Matlab Hospital [8]. The Dhaka Hospital is located at icddr,b’s headquarter in Dhaka, the capital city of Bangladesh. This hospital systematically enrolls 2% (every 50^th^) of all patients in the DDSS since 1996 [9]. The Matlab Hospital enrolls all diarrheal patients coming from its Health and Demographic Surveillance System (HDSS) villages in rural Matlab, about 55 kilometers south-west of Dhaka. The DDSS collects demographic, clinical and laboratory information from the enrolled patients and maintains electronic databases by using identical data collection tools. In this study, we collected relevant information from these databases to assess the proportion of typhoidal *Salmonella* i.e. *Salmonella enterica* Typhi and Paratyphi, and compared the differences in aspect of the proportion and socio-demographic, clinical and host characteristics, seasonality and antimicrobial susceptibility patterns.

## Methods

### Study design, source of data and sample framing

A case–control study design had been employed. Individuals with positive stool culture for TS, with other enteric co-pathogens such as *Vibrio cholerae, Shigella* spp., *Campylobacter*, *Escherichia coli*, rotavirus, *Entamoeba histolytica,* and *Giardia lamblia* were considered as case irrespective of age, sex and socio-economic status*.* Randomly selected diarrheal patients without TS constituted the controls.

For this 13 year period (2000–2012) analyses, we extracted the relevant information from the data archive of DDSS of both urban Dhaka and rural Matlab Hospitals. During the study period, a total of 25,767 patients in Dhaka and 17,622 in Matlab Hospital were enrolled in the surveillance system and 77 (0.30%) and 290 (1.65%) of them respectively has a positive stool culture for TS, including all other enteric co-pathogens (detailed distribution of these co-pathogens is listed in Table 1). These 77 and 290 patients constituted the case study population. Comparison groups were randomly selected as 1:3 (control) ratio to increase the power of the present analysis. Thus 231 and 870 individuals from Dhaka and Matlab were considered as comparison groups as diarrhea patients negative for TS. The patients of either sex, segregated into two age strata: 0 to 14 years of age and 15 years and above. Those who attended the hospital between the study periods were randomly selected as selection of age stratified controls.

**Table 1:** **Distribution of co-infection with Typhoidal** ***Salmonella*** **and pathogens among diarrhea patients negative for TS**

Pathogens	Co-infection with TS		Pathogen among diarrhea patients negative for TS	
	Dhaka; n = 77 (%)	Matlab; n = 290 (%)	Dhaka; n = 231 (%)	Matlab; n = 870 (%)
*Vibrio cholerae* 01	2 (3)	8 (3)	62 (27)	100 (8)
*Vibrio cholerae* 0139	0	0	2 (1)	7 (1)
*Vibrio cholerae non* 01	0	11 (4)	0	27 (3)
*Shigella* spp.	0	10 (3)	11 (5)	86 (10)
*Campylobacter* spp.	2 (3)	6 (2)	9 (4)	9 (1)
*Aeromonas* spp.	2 (3)	ND	8	ND
Rotavirus	10 (13)	28 (10)	55 (24)	123 (14)
*Giardia lamblia*	0	4 (1)	6 (3)	25 (3)
*Entamoeba histolytica*	0	3 (1)	1 (0.4)	6 (1)

ND: not done.

### Lab methodology

All patients coming from HDSS area in Matlab were included and their stool specimens were processed in the Matlab Microbiology Laboratory; a 2% sub sample from Dhaka Hospital were examined in the central laboratory in Dhaka. Each specimen was aliquoted into three serial containers and submitted to the respective laboratories for routine screening of common enteric pathogens such as *Salmonella* spp. [10], *Vibrio cholerae*[11], *Shigella* spp. [10], *Campylobacter*[10], *Escherichia coli*[12], rotavirus [13], *Entamoeba histolytica*[10]*,* and *Giardia lamblia*[10], isolated and identified using standard laboratory methods throughout the study period in both the site*.*

Susceptibility to antimicrobial agents was determined by the disk diffusion method as recommended by the Clinical Laboratory Standards Institute (CLSI 2010, June update) with commercial antimicrobial discs (Oxoid, Basingstoke, United Kingdom). The antibiotic discs used were; ampicillin (10 μg), nalidixic acid (30 μg), choleramphenecol (30 μg), trimethoprim-sulfamethoxazole (TMP-SXT) (25 μg), ciprofloxacin (5 μg), and ceftriaxone (30 μg) [14].

### Data analysis

Data were analyzed using Statistical Package for Social Sciences (SPSS) Windows (Version 15.2; Chicago, IL) and Epi Info (Version 6.0, USD, Stone Mountain, GA). Proportional differences were compared by Chi-square test, and assessed strength of association by estimating odds ratios (OR) and 95% confidence intervals (CI) around them. Two-sided alpha (probability) of <0.05 was considered to be statistically significant. Nutritional status (z-score for children under-5) was calculated using WHO-anthro 2005 software and malnutrition was defined as WHO guideline as < -2.00 SD [underweight (weight-for-age z-score), stunting (height-for-age z-score), wasting (weight-for-height z-score)] [15]. Site specific multivariate logistic regression analyses were also performed to determine the predictors of TS and variables identified to be independently associated (p < 0.05) (univariate analysis) with the outcome variable in each model. Variables which were age specific, for example malnutrition (underweight, stunting and wasting) among under-5 children, maternal and paternal education upto 14 years of age were not included, due to harmonizing the analysis in both the sites.

Two poisson regression models were investigated for modelling seasonal effects on the number of cases [16]**.** Firstly, a cosinor poisson regression model with year effects was fitted to both the monthly and daily time series at each site. Residual plots and diagnostics showed no evidence of autocorrelation. Both monthly and daily data revealed the same patterns with the effects having similar p-values so only the monthly models are presented here. Secondly, generalised additive models (GAM) with 5 and 6 df per year were fitted to the daily time series at each site. However, while the model fits had similar AICs (Matlab: cosinor AIC = 2088, GAM on 40 df AIC = 2082 and Dhaka: cosinor AIC = 797, GAM on 30 df AIC = 808), these models suffered from convergence problems and exhibited significant autocorrelation. Hence the simpler and more parsimonious cosinor models are presented. The models and residual diagnostics were carried out using the *R season* package [16]**.** The monthly model was slightly over dispersed for Matlab due to excess zeros (Matlab: residual deviance *X*^2^ = 192.3 on 141 df, Dhaka: 145.7 on 141 df) since we would expect the *X*^2^ to be about equal to the df if there was no over dispersion.

### Ethical consideration

The DDSS of icddr,b is a routine activity of the Dhaka and Matlab Hospitals, which have approval of both the Research Review Committee and Ethical Review Committee of icddr,b. Accordingly, verbal consent was taken from the adult patients and from the parents/attending caregivers of the minors before their enrollment in the DDSS.

## Results and discussion

Of all TS isolates, *S.* Typhi [57% (n = 44) vs. 5% (n = 12); p < 0.001] was higher in Dhaka as compared to Matlab site. Whereas, *S.* Paratyphi (A and B) [96% (n = 278) vs. 43% (n = 33); p < 0.001] was more frequently isolated in the Maltab in contrast to Dhaka population.

Of all TS isolates from Dhaka individuals 0–14 years old, *S.* Typhi represented 67% of the isolates (n = 26/39) and *S.* Paratyphi represented 33% (n = 13/39). Conversely, for Maltab it was 4% (n = 5/116) and 96% (n = 111/116) respectively. Among 15 years and above individuals, the prevalence was 47% (n = 18/38) and 53% (n = 20/38) in Dhaka, and 4% (n = 7/174) and 96% (n = 167/174) in Maltab respectively.

Half of the TS positive individuals were aged 0–14 years in Dhaka and overall 56% were male. A significantly higher proportion of individuals with TS had vomiting, some to severe dehydration, fever, duration of diarrhea more than 1 day before admission to the hospital, use of antimicrobials at home, and presence of fecal red blood cells compared to their control group. However, a lower proportion of them needed to use intravenous saline for rehydration. A higher proportion were malnourished (underweight, stunting and wasting) (Table 2). In the multivariate analysis, after controlling for the confounders, TS gastroenteritis was significantly associated with fever, use of antimicrobials at home, and fecal red blood cells in Dhaka (Table 2).

**Table 2:** **Characteristics and stool microscopic examination findings among individuals of TS gastroenteritis with diarrhea patients negative for TS in urban Dhaka (2000–2012)**

Characteristic	TS n = 77 (%)	Diarrhea patients negative for TS n = 231 (%)	Unadjusted OR (95% CI) p value	Adjusted OR (95% CI) p value
Male sex	43 (56)	129 (56)	1.00 (0.58, 1.74) 0.8	-
Age stratum				-
0-14 years	39 (51)	117 (51)	1.00 (0.58, 1.73) 0.8	-
15 years and above	38 (49)	114 (49)	-	-
Maternal illiteracy	21/39 (54)	35/117 (30)	2.73 (1.22, 6.15) 0.012	-
Paternal illiteracy	19/39 (49)	40/117 (34)	1.83 (0.82, 4.07) 0.1	-
Poor socio-economic status (median monthly family income < US$115)	46 (60)	119 (52)	1.40 (0.80, 2.44) 0.2	-
Use of non-sanitary toilet	23 (30)	0 (0)	-	-
H/o abdominal pain	35 (46)	100 (43)	1.09 (0.63, 1.89) 0.8	-
H/o vomiting in the last 24 hours	63 (82)	164 (71)	1.84 (0.93, 3.70) 0.085	-
Fever (Temperature ≥37.8°C)	13 (17)	9 (4)	5.01 (1.90, 13.41) <0.001	5.86 (2.16, 15.94) 0.001
Watery stool (lack of mucus/blood)	74 (96)	219 (95)	1.35 (0.34, 6.21) 0.7	-
Dehydration (moderate/severe)	52 (68)	149 (65)	1.14 (0.64, 2.06) 0.7	-
Duration of diarrhea (>1 day)	50 (65)	105 (46)	2.22 (1.26, 3.93) 0.004	1.27 (0.66, 2.46) 0.5
Hospital stay of ≥24 hours	31 (40)	84 (36)	1.18 (0.67, 2.07) 0.6	-
Use of IV rehydration	19 (25)	92 (40)	0.49 (0.27, 0.92) 0.023	0.72 (0.36, 1.46) 0.368
Use of antimicrobial at home	58 (75)	85 (37)	5.24 (2.82, 9.81) <0.001	5.08 (2.60, 9.90) <0.001
Wasting (<5 years)	13/30 (43)	22/103 (21)	2.82 (1.09, 7.27) 0.030	-
Stunting (<5 years)	16/30 (53)	26/103 (25)	3.16 (1.27, 7.90) 0.011	-
Underweight (<5 years)	21/30 (70)	34/103 (33)	4.74 (1.82, 12.62) 0.006	-
Fecal red blood cell/hpf (1 to >50)	33 (43)	67 (29)	1.84 (1.04, 3.24) 0.035	2.53 (1.38, 4.64) 0.003
Fecal leukocytes/hpf (11 to >50)	37 (48)	96 (42)	1.30 (0.75, 2.26) 0.3	-
Fecal macrophage/hpf (1 to 10)	22 (29)	45 (20)	1.65 (0.88, 3.11) 0.1	-

**Figure 1:**
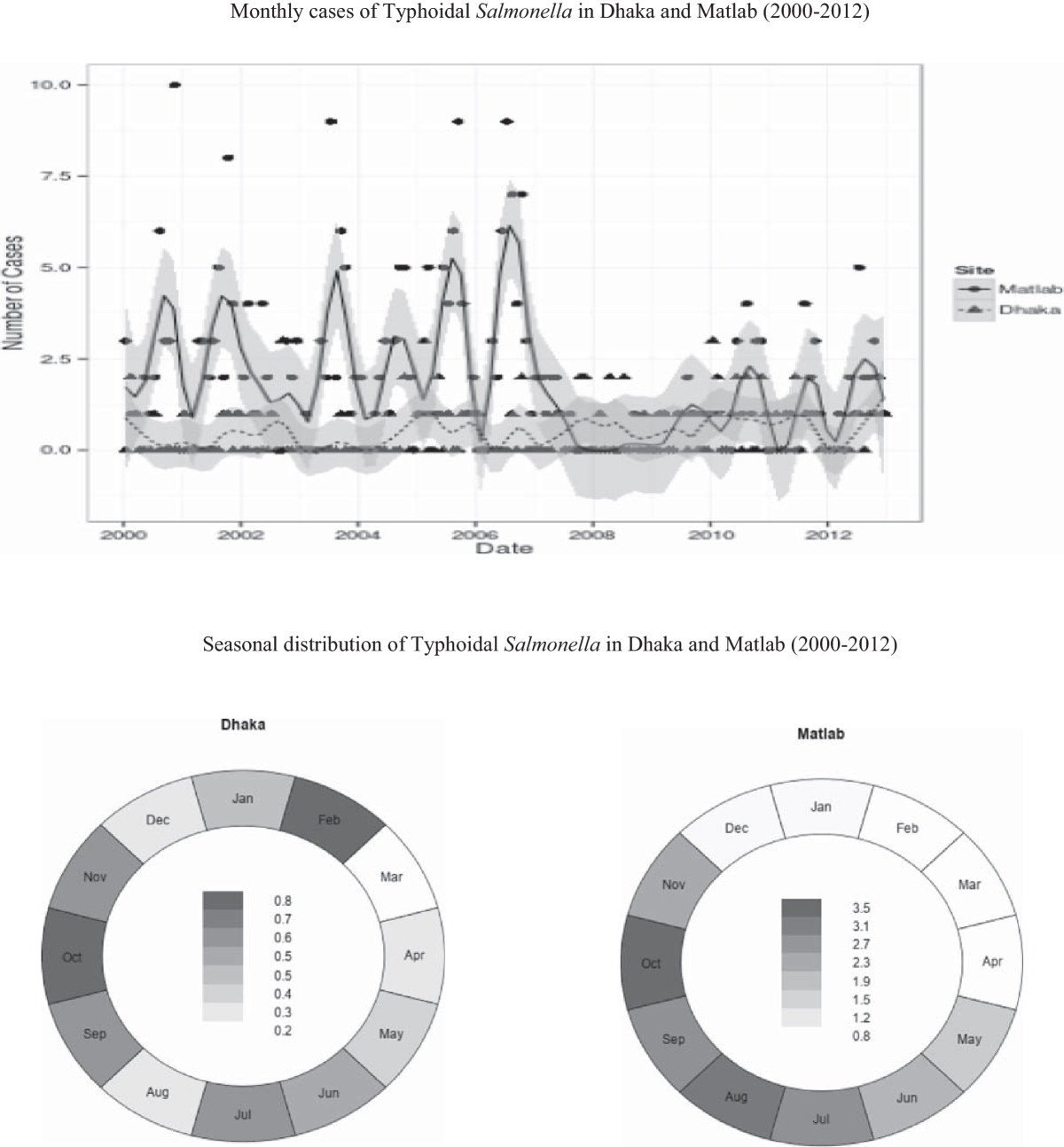
**Time trend and seasonal distribution of Typhoidal** ***Salmonella*** **in Dhaka and Matlab (2000–2012).**

In Matlab, a significantly higher proportion of TS individuals had vomiting, fever, longer duration of diarrhea (more than 1 day before admission to the hospital), use of antimicrobials at home, and higher number of fecal red blood cells, leucocytes and macrophage compared to control group. However, they needed less intravenous saline compared to Dhaka and exhibited a lesser number of red blood cells in stool (Table 3). In multivariate analysis, only fever, vomiting, longer duration of diarrhea, use of antimicrobials and fecal macrophage at home remained significantly associated with TS in addition to poor socioeconomic status (Table 3).The monthly data clearly exhibits a significant seasonal trend for Matlab and a significant difference between years (p < 0.001 for all effects). Dhaka does not show significant year or seasonal effects (p = 0.07 for years and p = 0.81 and p = 0.18 for the cos and sin components, respectively). Two seasonal peaks of TS were observed in Dhaka- during the months of January and February, and August to November in Dhaka, but a single peak in Matlab that corresponded to the later peak in Dhaka field site (Figure 1) but these were not significant.

**Table 3:** **Characteristics and stool microscopic examination findings among individuals of TS gastroenteritis with diarrhea patients negative for TS in rural Matlab (2000–2012)**

Characteristic	TS n = 290 (%)	Diarrhea patients negative for TS n = 870 (%)	Unadjusted OR (95% CI) p value	Adjusted OR (95% CI) p value
Male sex	161 (56)	440 (51)	1.22 (0.93, 1.61) 0.1	-
Age stratum				-
0-14 years	116 (40)	348 (40)	1.00 (0.76, 1.32) 0.9	-
15 years and above	174 (60)	552 (60)	-	-
Maternal illiteracy	25/116 (22)	65/348 (19)	1.20 (0.69, 2.07) 0.5	-
Paternal illiteracy	27/116 (23)	84/348 (24)	0.95 (0.56, 1.61) 0.9	-
Poor socio-economic status (median monthly family income < US$115)	168 (58)	467 (54)	1.19 (0.90, 1.57) 0.2	-
Use of non-sanitary toilet	245 (85)	0 (0)	<0.001 (0.00) <0.001	-
H/o abdominal pain	158 (55)	440 (51)	1.17 (0.89, 1.54) 0.2	-
H/o vomiting in the last 24 hours	219 (76)	570 (66)	1.62 (1.19, 2.22) 0.002	1.88 (1.35, 2.64) <0.001
Fever (Temperature ≥37.8°C)	100 (35)	128 (15)	3.05 (2.22, 4.19) <0.001	22.90 (2.08, 4.06) <0.001
Watery stool (lack of mucus/blood)	237 (82)	692 (80)	1.15 (0.81, 1.64) 0.4	-
Dehydration (moderate/severe)	152 (52)	417 (48)	1.20 (0.91, 1.58) 0.2	-
Duration of diarrhea (>1 day)	155 (53)	317 (36)	2.00 (1.52, 2.64) <0.001	1.78 (1.30, 2.44) <0.001
Hospital stay of ≥24 hours	148 (51)	407 (47)	1.19 (0.90, 1.56) 0.2	-
Use of IV rehydration	50 (17)	210 (24)	0.65 (0.46, 0.93) 0.018	0.74 (0.50, 1.09) 0.124
Use of antimicrobial at home	158 (55)	211 (24)	3.74 (2.88, 4.99) <0.001	3.15 (2.33, 4.27) <0.001
Wasting (<5 years)	28/95 (30)	73/319 (23)	1.41 (0.82, 2.42) 0.2	-
Stunting (<5 years)	31/95 (33)	101/319 (32)	1.05 (0.62, 1.75) 0.9	-
Underweight (<5 years)	32/95 (34)	125/320 (40)	0.79 (0.48, 1.32) 0.4	-
Fecal red blood cell/hpf (1 to >50)	207 (71)	508 (58)	1.78 (1.32, 2.40) <0.001	1.11 (0.67, 1.82) 0.7
Fecal leukocytes/hpf (11 to >50)	241 (83)	630 (72)	1.87 (1.31, 2.68) <0.001	1.44 (0.87, 2.39) 0.2
Fecal macrophage/hpf (1 to 10)	151 (52)	285 (33)	2.23 (1.69, 2.95) <0.001	1.89 (1.29, 2.74) 0.001

**Figure 2:**
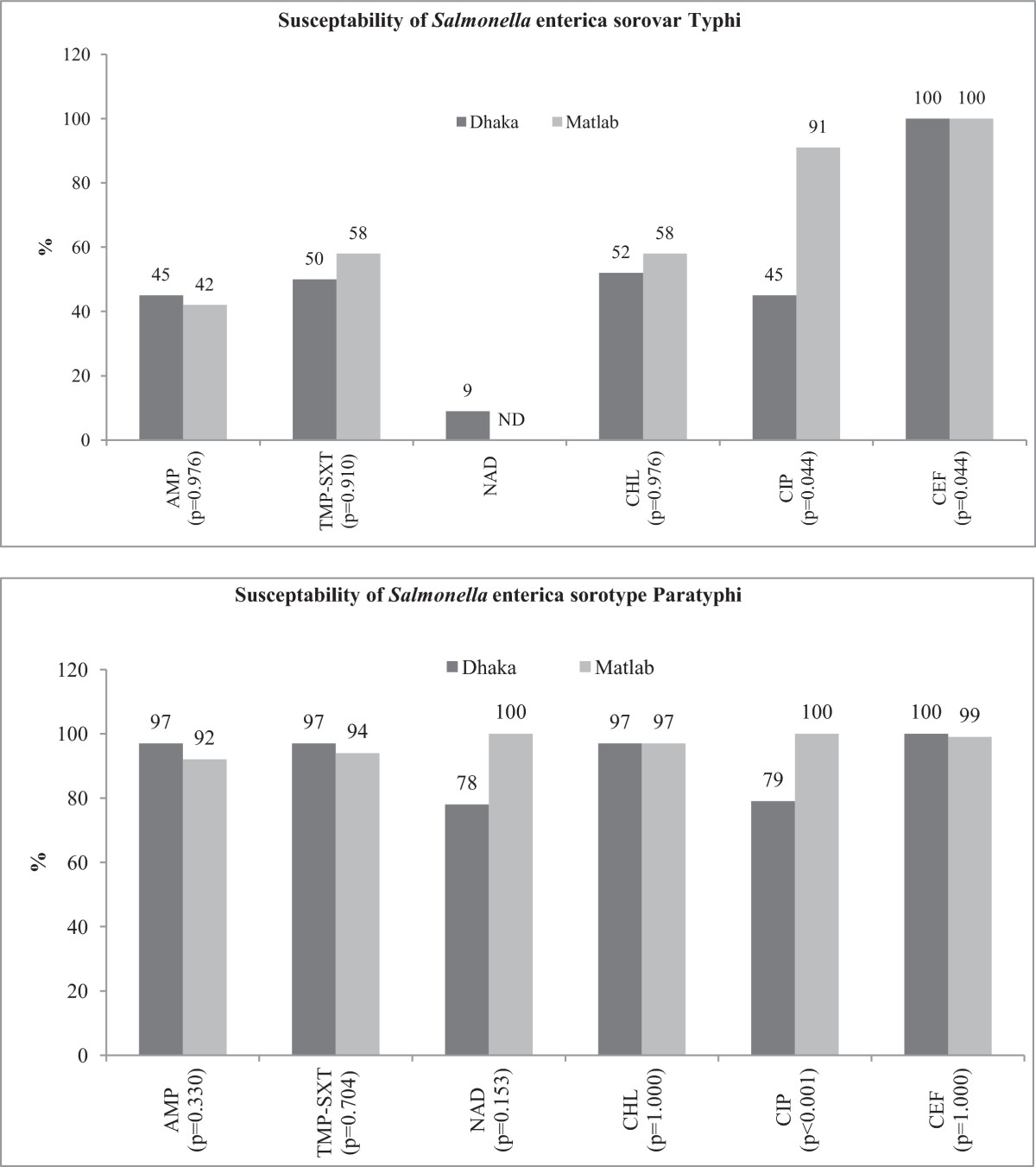
**Antimicrobial susceptibility of Typhoidal** ***Salmonella*** **in urban and rural sites (2000–2012).**

All *S.* Typhi isolates were susceptible to ceftriaxone in Dhaka and Matlab. For ciprofloxacin it was 45% vs. 91% respectively, while at least 52% of the isolates were susceptible to chloramphenicol at both sites. Susceptibility to ampicillin, trimethoprim-sulfamethoxazole and nalidixic acid ranged from 12% to 58% in the study areas (Figure 2). On the other hand, for *S.* Paratyphi, 99% were susceptibility to ceftriaxone in both the sites. Susceptibility patterns of other antimicrobials were shown in Figure 2.

Given the absence of information in medical literature, we assumed that the clinical and socio-demographic features of TS associated gastroenteritis might not differ by place of residence – urban Dhaka and rural Maltab as well as diarrhea due to other pathogens. However, we observed a number of important differences, which are likely to have public health implications. Among the differences, the prevalence of *S.* Typhi was higher and that of Paratyphi was lower in Dhaka compared to Matlab.

There are differences between Dhaka and Matlab populations with regards to food, water and hygiene practices. They all have significant associations with gastroenteritis irrespective of age, sex and socio-demographic contexts [17]. Most of the recent studies have described the features of gastroenteritis due to NTS but only a few described urban–rural (Dhaka-Matlab) differentials [7]. The present study might be the novel one to describe geographical heterogeneity of TS gastroenteritis irrespective of their age stratum. Increasing trend of *S.* Paratyphi was reported in rural areas and mostly associated with poor sanitation and socio-economic condition with lack of safe drinking water [18, 19]. Although, tube-well is considered as a safe source of drinking water in rural Matlab, sanitation facility in this rural catchment area is still very poor; this might explain the increased isolation rate of *S.* Paratyphi.

It was observed that, higher illiteracy rates among parents of Dhaka children with TS gastroenteritis. Literacy is an important factor for child care, proper feeding and sanitation practices including health care seeking behavior, leading to higher rates of malnutrition (underweight) in children from Dhaka than their Matlab counterparts [20]. This finding correlated with presence of vomiting with more dehydrating diarrhea [20]. On the other hand, malnutrition-associated altered immune status [21] might predict the susceptibility of infection with *Salmonella*. A high proportion of patients attending the Dhaka Hospital had received antimicrobials at home, which could explain higher rates of resistance to more commonly used oral antimicrobials relative to Matlab; access to health facilities and pharmacy in Dhaka may explain higher use and higher resistance. Lesser use of non-sanitary toilets is likely related to the availability of modern sewerage system, although contamination of supply water by sewerage leaks is believed to be common which may lead to outbreaks for enteric infections.

Presence of fecal inflammatory cells is an expected finding due to invasive nature of the pathogens*. Salmonella* is usually transmitted by fecal-oral route due to consumption of contaminated food or drinks, and organisms invade the intestinal mucosa leading to a disease by interaction with reticulo-endothelial system [22]. All of them were found statistically significant for Matlab model. However, only fecal red blood cell was significant for Dhaka. It might be due to the virulence properties of the pathogen or relative compromised nutrition among rural patients may explain better host defense including better inflammatory response and higher number of inflammatory cells. This is supported by higher proportion of Matlab individuals presenting with fever and their longer hospitalization.

An urban–rural differential was also observed in seasonality. There were rising seasonal effects at Matlab (p < 0.001) but no significant effects were seen at Dhaka. In addition to a common seasonal peak later in the year, a peak was also observed in urban Dhaka early in the year of winter months of January and February. Seasonal upsurges of pathogen is well documented elsewhere for other common diarrheal pathogens such as rotavirus [23], *Vibrio cholerae*[24] or *Shigella*[25, 26]. Several environmental as well as host characteristics may explain underlying predictions [27, 28]. Moreover, for Matlab, there appears to be a significant decrease around 2007 and we do not have any ready explanation for such reduction.

### Limitations

The present study was conducted among people attending diarrheal disease facilities, and may not be representative of the general population. The number of cases in Dhaka was very small which resulted in a diminished ability to detect seasonal differences. For Matlab, while more cases were seen, a high proportion of zeros was observed. While this caused convergence problems for the GAM model a cosinor poisson regression model with year effects fitted the data reasonably well and so zero inflated possion models were not necessary. However; in both the facilities, cost-free treatment is provided and it is accessible to all people, irrespective of their socioeconomic or other background, increasing the likelihood of including individuals with low socioeconomic status. Other strengths were the systematic unbiased sampling, large sample size of the study participants from surveillance system, and high quality laboratory performance further added to the strengths.

## Conclusion

Distinct geographical variations were observed with regard to socio-demographic and clinical characteristics among individuals with gastroenteritis due to TS between urban and rural areas.

## Authors’ original submitted files for images

Below are the links to the authors’ original submitted files for images. Authors’ original file for figure 1 Authors’ original file for figure 2

## Abbreviations

AMP: Ampicillin
CEF: Ceftriaxone
CHL: Chloramphenocol
CI: Confidence intervals
CIP: Ciprofloxacin
DDSS: Diarrhoeal disease surveillance system
HDDS: Health and demographic surveillance system
icddr: b: International centre for diarrhoeal disease research, Bangladesh
ND: Not done
NAD: Nalidixic acid
NTS: Non-typhoidal *Salmonella*
OR: Odds ratios
SPSS: Statistical package for social sciences
*S.* Typhi *Salmonella enterica*: serover Typhi
S. Paratyphi: *Salmonella enterica* serover and Partyphi
TMP-STX: Trimethoprim-sulfamethoxazole
TS: Typhoidal *Salmonella*.
