# Supplementary material for: Gastroenteritis due to typhoidal Salmonella: a decade of observation at an urban and a rural diarrheal disease hospital in Bangladesh
Source: BMC Infect Dis. 2014 Aug 7;14:435. doi: 10.1186/1471-2334-14-435 (PMC4132926; doi:10.1186/1471-2334-14-435)

Monthly cases of Typhoidal *Salmonella* in Dhaka and Matlab (2000-2012)

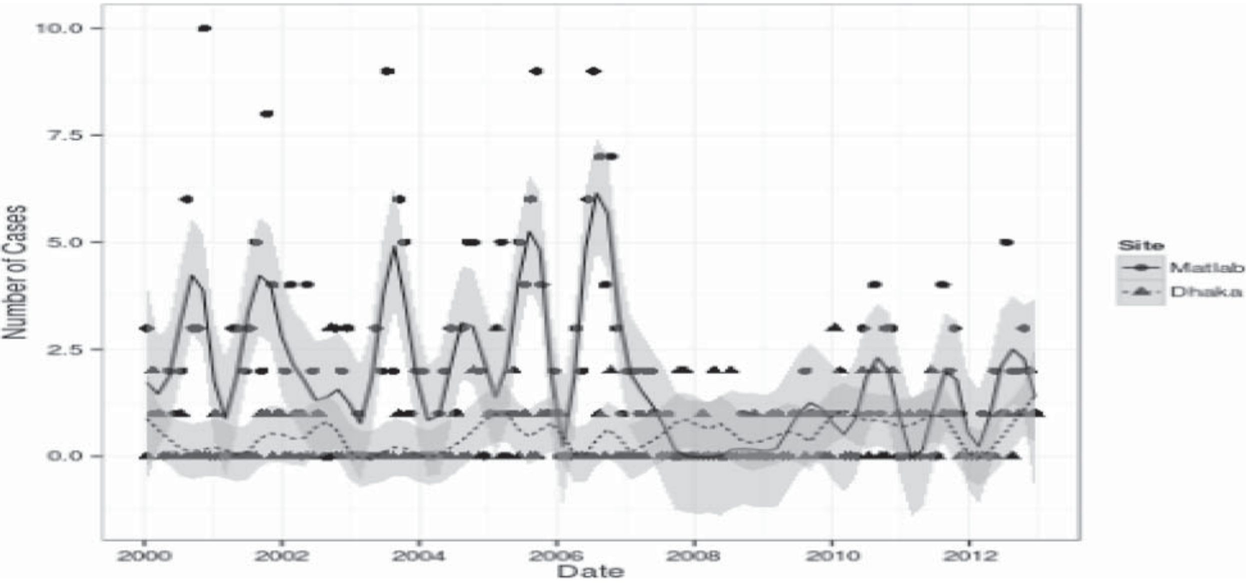

Seasonal distribution of Typhoidal *Salmonella* in Dhaka and Matlab (2000-2012)

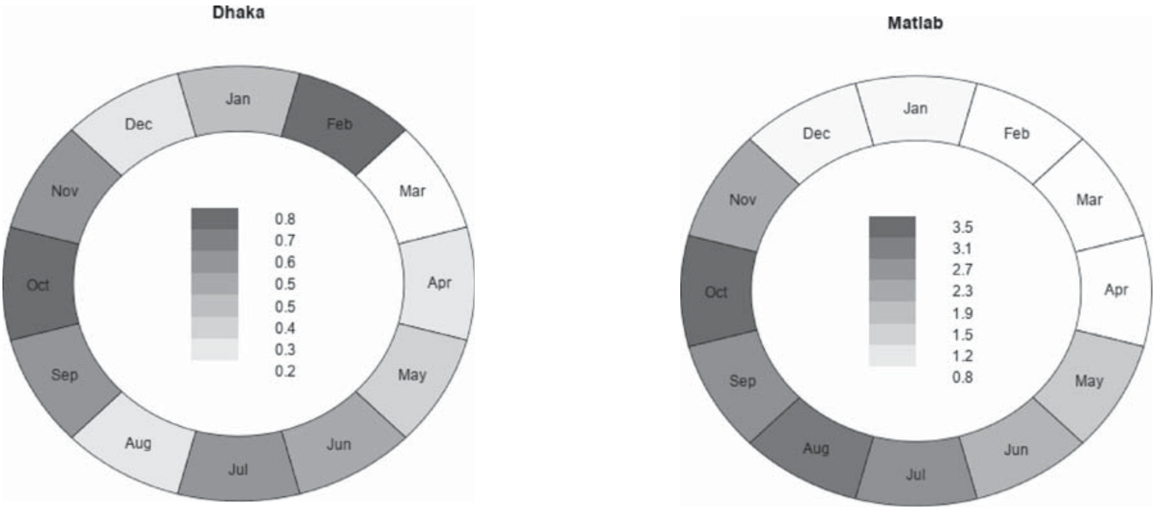

Supplement: Supplementary file 1 — Authors’ original file for figure 1 [file 12879_2013_3733_MOESM1_ESM.pdf]
