# Supplementary material for: Gastroenteritis due to typhoidal Salmonella: a decade of observation at an urban and a rural diarrheal disease hospital in Bangladesh
Source: BMC Infect Dis. 2014 Aug 7;14:435. doi: 10.1186/1471-2334-14-435 (PMC4132926; doi:10.1186/1471-2334-14-435)
Supplement: Supplementary file 2 — Authors’ original file for figure 2 [file 12879_2013_3733_MOESM2_ESM.pdf]

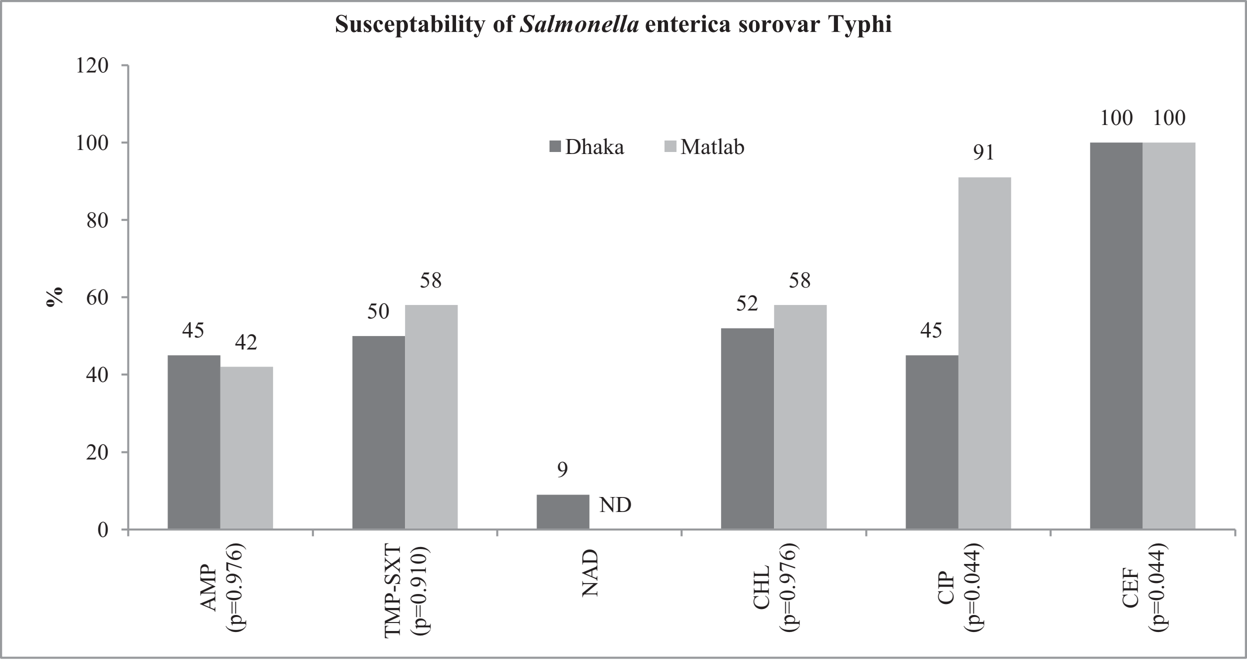

Susceptability of Salmonella enterica sorovar Typhi
Dhaka
Matlab
%
120
100
80
60
40
20
0
45
42
50
58
9
ND
52
58
45
91
100
100
AMP (p=0.976)
TMP-SXT (p=0.910)
NAD
CHL (p=0.976)
CIP (p=0.044)
CEF (p=0.044)

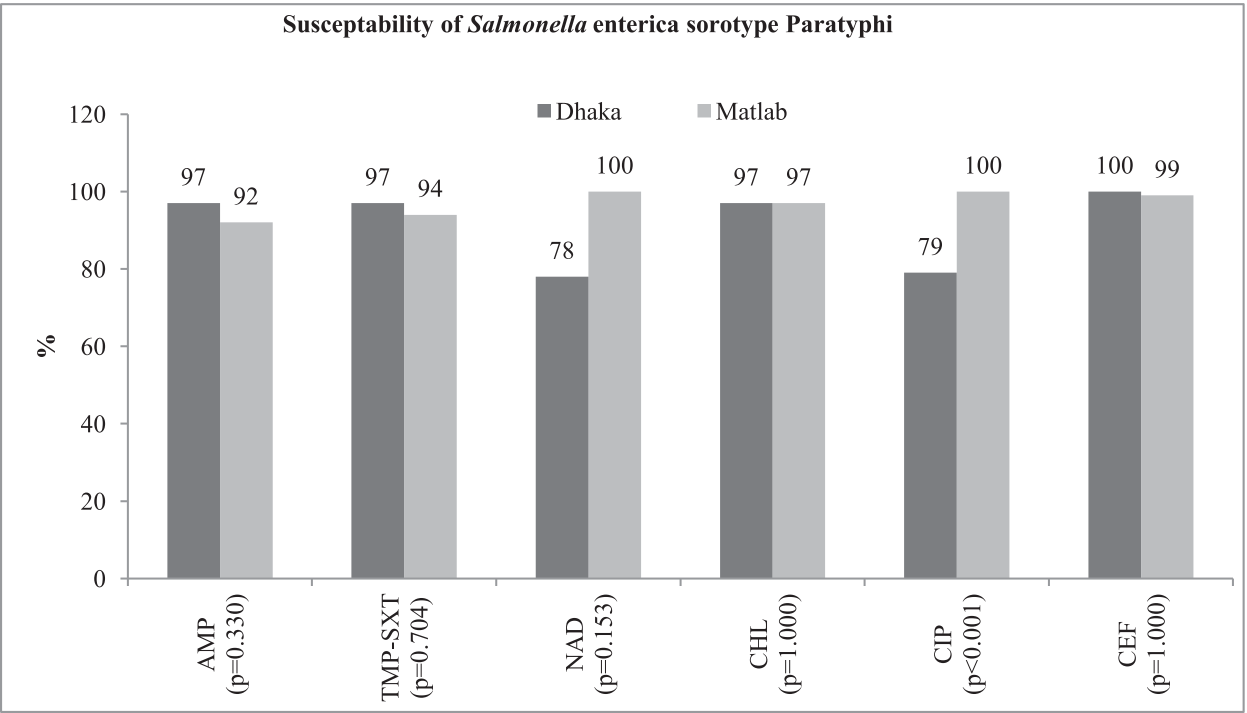

Susceptability of Salmonella enterica sorotype Paratyphi
Dhaka
Matlab
%
120
100
80
60
40
20
0
97
92
97
94
78
100
97
97
79
100
100
99
AMP (p=0.330)
TMP-SXT (p=0.704)
NAD (p=0.153)
CHL (p=1.000)
CIP (p<0.001)
CEF (p=1.000)
